# Stability Study of Algerian* Nigella sativa* Seeds Stored under Different Conditions

**DOI:** 10.1155/2017/7891434

**Published:** 2017-02-01

**Authors:** Muhammad Safwan Ahamad Bustamam, Kamarul Arifin Hadithon, Ahmed Mediani, Faridah Abas, Yaya Rukayadi, Nordin Lajis, Khozirah Shaari, Intan Safinar Ismail

**Affiliations:** ^1^Laboratory of Natural Products, Institute of Bioscience, Universiti Putra Malaysia, 43400 Serdang, Selangor, Malaysia; ^2^Department of Food Science, Faculty of Food Science and Technology, Universiti Putra Malaysia, 43400 Serdang, Selangor, Malaysia

## Abstract

In a study to determine the stability of the main volatile constituents of* Nigella sativa *seeds stored under several conditions, eight storage conditions were established, based on the ecological abiotic effects of air, heat, and light. Six replicates each were prepared and analyzed with Headspace-Gas Chromatography-Mass Spectrometry (HS-GC-MS) for three time points at the initial (1st day (0)), 14th (1), and 28th (2) day of storage. A targeted multivariate analysis of Principal Component Analysis revealed that the stability of the main volatile constituents of the whole seeds was better than that of the ground seeds. Exposed seeds, whole or ground, were observed to experience higher decrement of the volatile composition. These ecofactors of air, heat, and light are suggested to be directly responsible for the loss of volatiles in* N. sativa *seeds, particularly of the ground seeds.

## 1. Introduction

The biological activities of plants are due to the individual and synergistic and antagonistic effects of their distinct chemical compositions [1]. The chemical matrix of a plant part will vary dramatically depending on the plant genetic makeup and/or a range of environmental factors [2], including the steps during and after harvesting [3]. A recent study of four different origins of* Nigella sativa* L. (Ranunculaceae) seeds in relationship to three biological activities showed the differences between the seeds and their exhibited bioactivities [4]. Even though there is inherent variation within species (sometimes called “chemical races”) which is not controllable, the variations in chemical composition due to environmental changes could possibly be minimized, if the factors involved could be understood, leading to a more consistent product [5]. Extensive chemical and biological studies have been done on* N. sativa* seeds, and most of the pharmacological properties of the seeds and oil were attributed to the main volatiles content, in particular thymoquinone (1) which is the most abundant component (24-25%) [6–8].

According to SCIFinder statistical data until March 2011, many articles on* N. sativa* that were published involving numerous bioactive potentials, which include antihypertensive, antidiabetic, anticancer, gastroprotective, antioxidant, anti-inflammatory, antihistamine, antimicrobial, antitumor, antihepatotoxic, antinephrotoxic, respiratory, and immunomodulatory effects, have been conducted on* N. sativa* seeds [9]. Most of the these pharmacological activities of* N. sativa* were observed to be contributed by the main volatiles, particularly thymoquinone [10]. Due to these shown bioactivities of* N. sativa*, seed-based products mainly in health supplement formulation are widely available in the market. The shelf life and content consistency of the plant seeds and the oil are significantly affected by the stability of the volatiles and are thus important for the pharmacological effects of the seeds. The primary step in the efficacy and consistency of* N. sativa* seed-based product is the quality and safety assurance of the seeds. The primary care of seeds based on the suitable storage condition is crucial. Although this has been a concern on possible negative influence on the volatile constituents [5], no scientific study has been done to tackle this issue. Hence, Headspace-Gas Chromatography-Mass Spectrometry (HS-GC-MS), in combination with multivariate data analysis, enabled an assessment of the ecofactors effects of air, heat, and light, on the main volatile constituents of* N. sativa* seeds (Supplement Material 1, in Supplementary Material available online at https://doi.org/10.1155/2017/7891434) based on a real time storage of how the customer stored the seeds. This is in order to elucidate the guidelines for the optimum storage conditions.

## 2. Materials and Methods

### 2.1. Plant Material and Chemicals

The* Nigella sativa *seeds were purchased from a retail store in Adrar, Algeria, during the harvesting season in September 2012. The whole seeds sold in a gunny were packed in a vacuumed plastic bag and transported to Malaysia immediately after being purchased in December 2012. They were then stored in the chiller at 4°C after being identified by Dr. Shamsul Khamis, the botanist from the Institute of Bioscience (IBS), Universiti Putra Malaysia (UPM) under specimen voucher number SK 2518/14. Standard compounds D-carvone, thymoquinone, and carvacrol were purchased from Sigma/Aldrich (St. Louis, MO, USA). Longifolene was purchased from ChromaDex (Irvine, CA).

### 2.2. Storage Procedure

The seeds taken out from the chiller were kept in a vacuum desiccator overnight to ensure being moisture-free and dried until a constant weight was obtained. Samples consisting of 1 g seeds each were prepared in 10 mL screw top amber or clear vials (Agilent Technologies, CA, USA) with polypropylene hole cap and PTFE/silicone septa (Agilent Technologies, CA, USA). The vials were stored in eight different conditions of six replicates (*n* = 6) for each condition, for three time points of initial (1st), 14th, and 28th day. The samples were then analyzed with HS-GC-MS. For storage condition A, 1 g of the whole seeds which filled 1/3 of an amber vial was prepared. Storage condition B was an amber vial with fully filled whole seeds. Similar to condition B, C was also with the vial filled with the whole seeds, but in a transparent/clear vial. The D condition was similar to A; however, D was flushed with nitrogen gas for 30 minutes before being capped. For conditions E, F, and G, the whole seeds were ground in a stainless steel analytical mill grinder (IKA A11 Basic, Sigma-Aldrich, St. Louis, MO, USA) into a rough powder of 500 *μ*m. Conditions E, F, and G seeds were treated similarly as conditions A, B, and D, respectively. All samples of conditions A to G were stored in a closed dark cupboard at ambient temperature until the set time points. Sample H was prepared similar to A but differently from other samples as it was placed on the laboratory bench unhinderedly receiving emission of light (36 watt) for 10 hours (8.00 am–6.00 pm) throughout the storage time. All the prepared samples were tightly capped. The compositions of the major constituents of the seed samples under all storage conditions were analyzed using HS-GC-MS at three time points. The methodology is as presented in Supplementary Material 2.

### 2.3. Static Headspace Condition

Analysis of the major volatile compounds was performed using a static headspace technique involving a Thermo Scientific Trace GC Ultra gas chromatograph, in combination with a CTC Combi PAL autosampler and MS TSQ Quantum XLS mass selective detector. The samples were incubated for 600 s at 160°C with 250 ppm agitator speed; fill speed, 100 *μ*L/s; pressurizing time, 1.5 min; and flush time, 60 s. The sample loop and transfer line temperatures were set at 10 and 20°C higher than the oven temperature, respectively.

### 2.4. Gas Chromatography-Mass Spectrum (GCMS) Conditions

The compounds were separated in a fused-silica-capillary column with a nonpolar stationary phase Trace GOLD TG-5MS (Thermo Fisher Scientific Inc., USA) with specification of 30 m length; 0.25 mm ID; 1.4 *µ*m film thickness of 5% biphenyl and 95% dimethylpolysiloxane. The oven temperature was programmed at 60°C for 2 min, then progressed from 60 to 280°C at the rate of 5°C/min, and held at 280°C for 5 min. The temperatures for the injection port, ion source, triple quadrupole, and interface were set at 250, 230, 150, and 240°C, respectively. Mass spectra were obtained in the electron impact (EI) mode at 70 eV in a full scan of range from* m/z* 50 to 650. The scan rate was set at 3.95 scan/sec. The homologous series of C_7_–C_33_* n*-alkanes (10 *μ*L) was diluted in 0.5 mL HPLC grade hexane and 1 *μ*L of the dilution was then directly injected into the GC operating under the same conditions as described above.

### 2.5. Qualitative Analysis of Targeted Compounds

Identification of the targeted compounds was based on the comparison of the obtained sample spectral peak signals with those of the National Institute of Standards and Technology (NIST11) database library and with four out of six selected purchased standards: carvone, thymoquinone, carvacrol, and longifolene. Percent peak area for each compound was calculated by dividing its response area with all peaks areas detected in the entire MS spectrum. The results were further verified by comparison of linear retention indices (LRIs) of the identified compounds with the reference data [11] as shown in Table 1. Thymohydroquinone in this sample was identified as phenol,4-methoxy-2,3,6-trimethyl since the identity by the name of thymohydroquinone was not available in NIST11 library. The molecular weights and mass spectra of phenol,4-methoxy-2,3,6-trimethyl and thymohydroquinone are similar, even when compared to the data in Flavour and Fragrance Natural and Synthetic Compounds (FFNSC) version 1.3 library (Supplementary Material 6). Hence, the presence of thymohydroquinone is confirmed based on the identification of phenol,4-methoxy-2,3,6-trimethyl in the GCMS data of the samples. A few other compounds were putatively identified as there was no comparable data found in Adams libraries spectra [11].

### 2.6. Multivariate Analysis

A signal in the total ion chromatogram (TIC) representing metabolite content was calculated according to its peak area which was converted to a single data matrix. The data was imported into SIMCA-P version 13.0 (Umetrics, Umeå, Sweden) whereby the generated normalized metabolite peaks were transformed into variables. After the mean centering and UV scaling of the data, correlations among the samples were then established by the Principal Component Analysis (PCA) model. The analysis was validated by default sevenfold internal cross-validation based on the *R*^2^*X* and *Q*^2^ values which are the goodness of fit and goodness of prediction, respectively. The PCA, which is an unsupervised analysis, was conducted in order to determine an overview of the possible differences or similarities among the groups of samples based on the observation in the score plots [12].

### 2.7. Statistical Analysis

The peak areas of the selected main constituents were reported as mean ± standard deviation. The chromatographic analysis was done on six replicates (*n* = 6). Significant differences between the samples were evaluated using Minitab 14 (Minitab Inc., State College, PA, USA) performing one-way ANOVA test and a Turkey comparison test with a confidence interval of 95%.

## 3. Results and Discussion

### 3.1. Qualitative Analysis of Targeted Compounds

*N. sativa *seeds were previously shown to be rich in volatile constituents; however, the composition differs among the different geographical origin of the plants.* N. sativa* matured seeds collected in Ein-Harod, Israel, have been recognized to contain* p-*cymene (25%), thymohydroquinone (16.2%), *γ*-terpinene (11.6%), *α*-thujene (9.7%), thymoquinone (7.8%), and carvacrol (5.8%) as the main compositions of monoterpenes [13]. Thirty-eight constituents representing 84.6% of the total amount of Indian* N. sativa* essential oil obtained through hydrodistillation with major components of* p*-cymene (36.2%), thymoquinone (11.27%), *α*-thujene (10.03%), longifolene (6.32%), *β*-pinene (1.3%), and carvacrol (2.12%) were identified [14]. Benkaci-Ali et al. (2006) also reported the same major compounds of the Algerian* N. sativa* essential oil with the Indian originated seeds, but in different percentages after being extracted with microwave distillation, which were* p*-cymene (53.83%), thymoquinone (17.04%), *α*-thujene (11.91%), *β*-pinene (1.96%), *α-*longifolene (6.32%), and carvacrol (0.68%) [15]. These main constituents were widely reported to be responsible for most of the biological activities shown by* N. sativa *[6, 8, 16].

**Figure 1:**
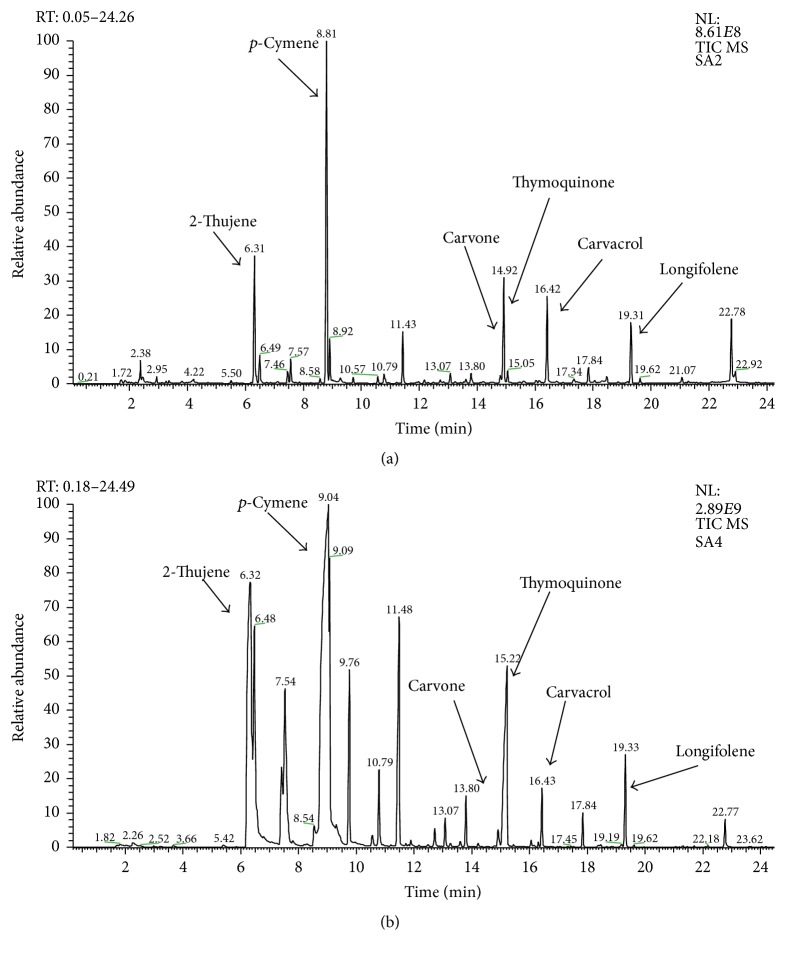
Total ion chromatogram (TIC) profile of whole* N. sativa *(a) and ground* N. sativa *(b).

In the present study, the TIC profiles of the whole and ground* N. sativa *seeds as displayed in Figure 1 indicate these two groups have a similar characteristic profile of compounds but varied in their concentrations. A total of twenty-five volatile secondary compounds were successfully identified in both seed forms, as shown in Table 1. The composition of each constituent was expressed in TIC area unit (10x^7^) and percentage (%). Most of the constituents detected could be grouped into monoterpenoid hydrocarbon and monoterpenoid ketone classes of compounds, as reported previously. However, the ground seeds exhibited higher concentrations of most of the major constituents compared to the whole seeds, suggesting that these volatiles were exposed upon crushing of the seeds. The most dominant constituents of the ground seeds were observed hierarchically to be* p*-cymene, 2-thujene, thymoquinone, longifolene, carvacrol, and carvone. Meanwhile, the whole seeds afforded* p*-cymene, thymoquinone, carvone, 2-thujene, carvacrol, and longifolene. These results are in agreement with the findings of Benkaci-Ali et al. (2006) [15]. Although the majority of the constituents were detected at a higher level in the ground seeds, carvone was found to be more dominant in the whole seeds. This probably due to the different compounds rate of volatility in which carvone might have higher volatility rate causing them to be immediately reduced when the seeds were crushed [17].

### 3.2. Multivariate Data Analysis of Each Storage Condition

**Figure 2:**
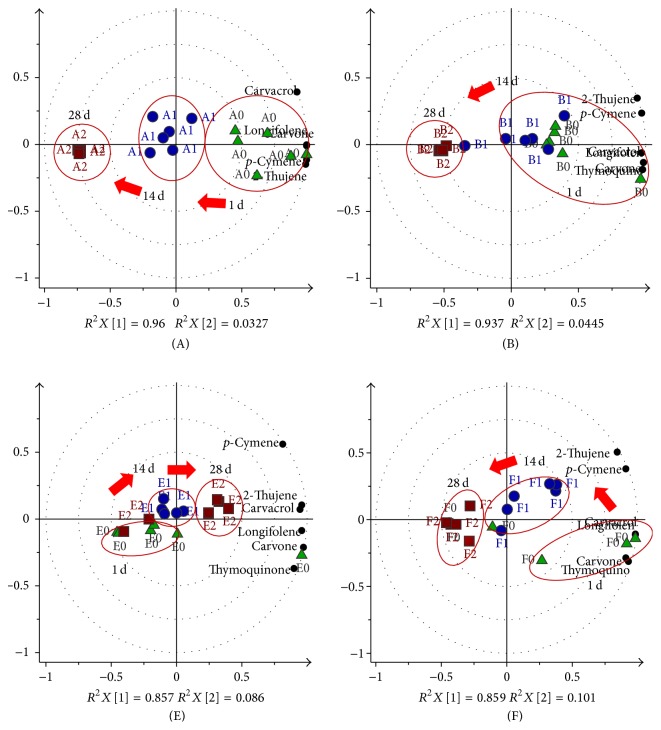
The biplot model of the principle component analysis (PCA) for storage to determine the effects of air on the seeds; 1st (green triangle), 14th (blue circle), and 28th (red square) day. The smaller black circles are those identified compounds from the GCMS.

**Figure 3:**
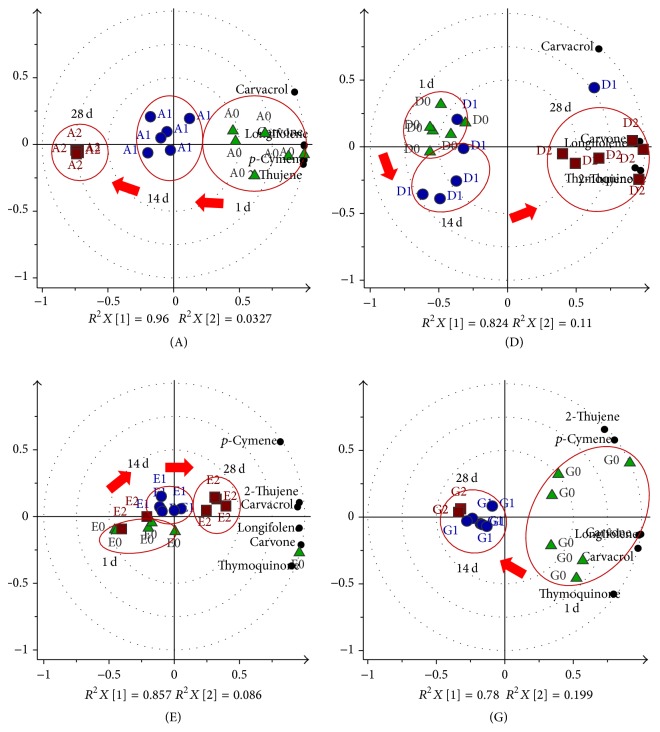
The biplot model of the principle component analysis (PCA) for storage to determine the effects of air; 1st (green triangle), 14th (blue circle), and 28th (red square) day. The smaller black circles are those identified compounds from the GCMS.

**Figure 4:**
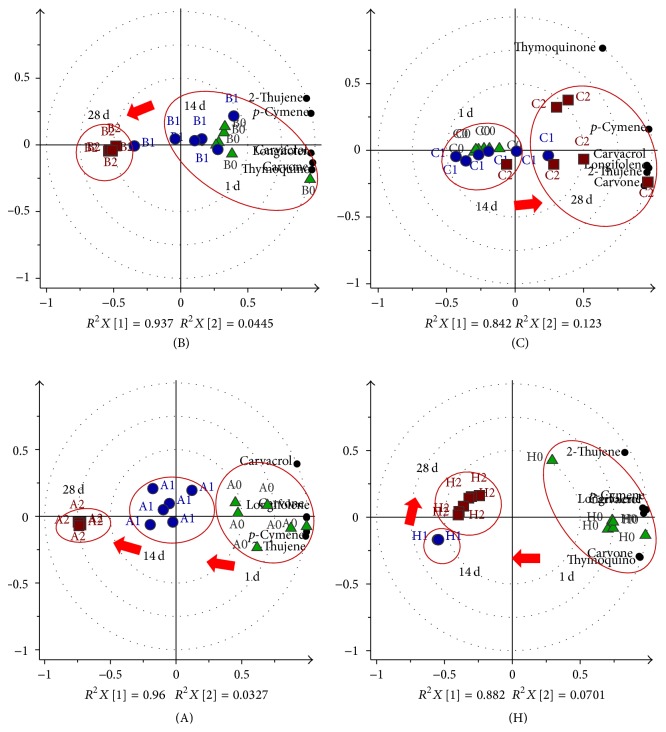
The biplot model of the principle component analysis (PCA) for storage to determine the effects of heat and light; 1st (green triangle), 14th (blue circle), and 28th (red square) day. The smaller black circles are those identified compounds from the GCMS.

The absolute peak areas of the major compounds, namely, 2-thujene,* p*-cymene, carvone, thymoquinone, carvacrol, and longifolene, as provided in Supplementary Material 3 were used as variables in the Principal Component Analysis (PCA). These variables were standardized and the dataset was centered and evaluated by cross-validation in the multivariate model. The stability of each storage condition (A–H) which dataset generated using UV scaling was statistically analyzed by PCA. Three storage time points (tp) of 1st day, 14th day, and 28th day were assigned as 0, 1, and 2 tp, respectively, to assist in the understanding of the discussion of results. The 0 tp in each condition was used as the control and compared with the other storage time points. The quality of each PCA model was evaluated by the cumulative values of *R*^2^*X* and *Q*^2^ which are the goodness of fit and predictive ability of a model, respectively. The model fitness and predictive ability are considered good when the *Q*^2^ and *R*^2^ values are greater than 0.5 and their differences are in the range of 0.2–0.3 [18]. All the PCA biplot models (Figures 2–4) in this study have exhibited good fitness and predictive features with *R*^2^*X* (cum) and *Q*^2^ (cum) scores between 0.933–0.994 and 0.709–0.952, respectively.

However, the retention time shifted by almost 1 minute in some samples, due to instrumental glitches, which made peak alignment difficult to be performed [19, 20]. Zhao et al. (2011) in their study suggested another approach, which is through the content of constituents summarized from the obtained TICs into a matrix in an Excel file [21]. This approach was then adopted by Ahmad et al. (2014) with some modifications, wherein the intensities of all the peaks from a spectrum of each sample were rearranged into one single table and transferred into Microsoft Excel 2007 [22]. The Excel file was then imported into SIMCA software (Umetrics, Umea, Sweden) for statistical analysis. This described method was effective and reliable, since any shifting of retention time due to the instrumental or other experimental condition differences, such as column cut, could be resolved. Hence, the current study analyzed the mass spectroscopic data based on the method done by Ahmad et al. (2014) [22].

#### 3.2.1. Effect of Air on the Seeds

Two storage conditions, A and B, were set to observe the effect of air on the seeds as displayed in Figure 2. Both of these conditions used whole seeds in amber vials, however with different amount of the seeds, wherein A and B were 1/3 and fully filled, respectively. The purpose of setting this condition is to investigate the influence of the available air inside the vial on the seed volatiles. The PCA plots for condition A exhibited clear separation between the three time points by principle component t[1] which explained 96% variation among the variables (*R*^2^*X*). The plot displayed the volatiles being more in the positive quadrant close to the initial day (0 tp) indicating a higher constituents content in this group discriminated from 1 tp and 2 tp groups which were on the negative side. This clustering pattern suggested a progressive decrease in concentrations about 29.7–52.2% of the six selected constituents after 14 days and more (84.97–90.79%) after 28 days. However, the plots of condition B showed 0 and 1 tp clustered together with the constituents in positive quadrant by PC1 with 93.7% variable variation from 2 tp, which suggested the longer maintenance of the volatiles compared to A. Less air in B as the seeds were packed fully in the vial probably assisted in reducing the loss of the volatile constituents. The clustering together of the 2 tp for both A and B in the opposite quadrant from the constituents supported that both of these storage conditions experienced the highest decrement of volatiles after 28 days of storage. The decreased levels of the main volatiles might be due to their autoxidation in the presence of oxygen. It was reported that the peroxide formed by autoxidation was high above 10 in* N. sativa* seed oil when exposed to open air or oxygen [23, 24]. It could be suggested that the air in the vial influenced the changes of the volatiles as reported in the previous study by Kalua et al. (2006) on the chemical properties between the virgin olive oil exposed to oxygen compared to that stored in the absence of oxygen [5].

The effect of air on the* N. sativa* ground seeds was also determined through conditions E and F (Figure 2), which were prepared similar to conditions A and B. However, the preparation of the ground seed samples was difficult to be standardized among the replicates since the volatiles were immediately exposed upon being crushed. Thus, the intravariation of the replicates was observed to be high. Condition E showed clear contradiction of trend when compared to A or B wherein the volatile concentrations from all replicates increased from 0 tp to 2 tp. The constituent compositions in E exhibited elevation in the range of 24.1–135%, except for carvone and thymoquinone as shown in Supplementary Material 3. These two compounds decreased by 30% and 12.2%, respectively, which is in agreement with the observation explained earlier in Section 3.1. Ahmad et al. (2013) reported that thymoquinone tends to form dithymoquinone and other higher oligocondensation products when stored [10]. Much similar to the observation in A, F also exhibited gradual reduction of the volatile concentrations after 1 tp. The volatile composition of F in the biplot shown in Figure 2 exhibited great decrement (80.1–92.9%) at the 2 tp. Although the ground seeds were fully packed in the vial with less air, volatiles tended to be lost when exposed through decapping as only 1 g of the packed seeds was transferred to be analyzed by HS-GCMS.

Another approach of looking at the effect of air was observed through the comparison of storage conditions A and D (Figure 3), wherein the only difference is that the latter was flushed with nitrogen gas for 30 minutes before being capped. This was done in order to remove as much as possible the air from the vial. The PCA plot of D shows 82.4% of the total spectral variation by principle component t[1] discriminating both 0 and 1 tp from 2 tp group with pattern of increment in the constituents of D which was contradictory to A. It seems that the seeds stored in air-flushed condition could preserve the volatiles and increase the retention of those constituents. This is in agreement with the reported study which stated that the removal of oxygen from the atmosphere contributed to reduction of aerobic microbial growth, some enzymatic reactions, and lipid oxidation [25, 26].

The effect of air on the ground seeds was also observed through condition G which was compared with E, as shown in Figure 3. By principle component t[1], the volatile compositions changes in G were very different from E in which G was relatively dominant in the initial group but experienced high decrement of 97.5–99.8% after 14 and 28 days of storage. This might probably be because the nitrogen gas has flushed out the volatiles during the sample preparation. Contrary to G, the whole seeds prepared in the similar manner showed an increase of the volatile content. Hence, the constant flushing of nitrogen gas for 30 minutes on the whole seeds before packing would be helpful in preserving those volatile constituents.

#### 3.2.2. Effects of Heat and Light

The influence of heat and light was also been studied by comparing the storage of the fully packed whole seeds in a transparent vial (condition C) with those in an amber vial (condition B), which were both stored in a closed dark cupboard (Figure 4). PCA plot of C contributed 96% of the total variance which exhibited the least separation between the initial and 14th day, but the compound concentrations increased by 1.2–2.7 times at the second time point. High intravariation among the 2 tp replicates suggested that the stability of the volatiles was probably significantly affected when the seeds were stored in the transparent vial. The sample in B as discussed in Section 3.2.1 exhibited the gradual loss of volatiles in time. The comparison results between these two conditions suggested that the transparent vial was better to be used for storage as the amber vial might tend to absorb more heat, which possibly caused substantial deterioration of quality and freshness. However, heat was observed to be giving less effect than light as seen in the study on olive oil [27].

The light effect was seen through the comparison of conditions H and A (Figure 4) whereby both of them were prepared with 1/3 filled whole seeds in amber vials. H was placed on the bench with 10 : 14 light/dark cycle while A was kept in the dark cupboard as described before. The PCA plot of H showed the separation of 0 tp from 1 and 2 tp clusters by principle component t[1] with 88% total spectral variation. The biplot for H displayed all of the constituents being concentrated at the initial time point in the positive quadrant of the t[1], while the other two time points were isolated away from the constituents. The volatile content was observed to be high in the first day but decreased remarkably of 97.6–99.4% after 14 days. Differently in A, in which the constituent decrement occurred slowly, the presence of light might have promoted abrupt degradation of the volatiles. Kalua et al. (2006) also found that all volatile compounds in fresh virgin olive oil decreased during storage under the light [5]. Thus, the storage of volatile constituents is preferable to be protected from light.

### 3.3. Multivariate Data Analysis between Storage Conditions

#### 3.3.1. Discrimination between the Storage Conditions A, B, C, and D for the Whole Seeds

**Figure 5:**
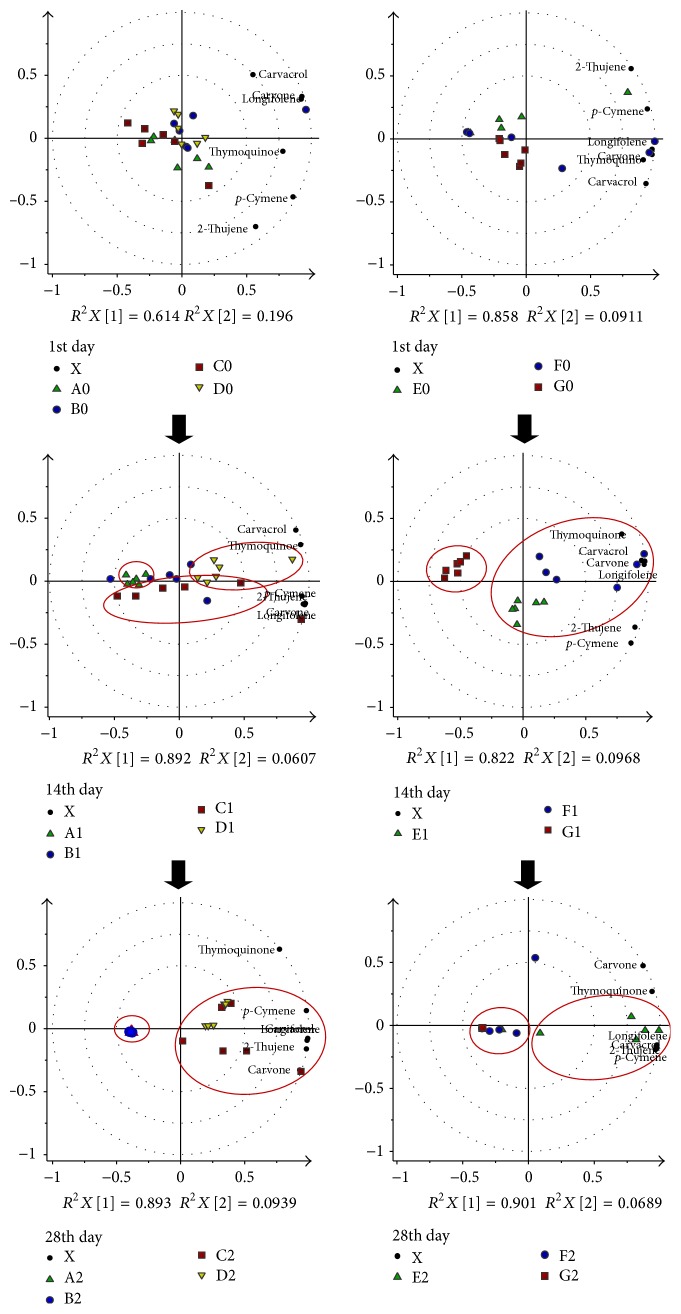
The biplot model of the principle component analysis (PCA) for the comparison of condition A (green triangle), B (blue circle), C (red square), and D (yellow triangle) for whole seeds (left) and condition E (green triangle), F (blue circle), and G (red square) for ground seeds (right) along the storage period. The blue circle refers to the time point of 14th (2). The smaller black circles are those identified compounds from the GCMS.

The comparison between the storage conditions of the whole seed samples A, B, C, and D is displayed in Figure 5 (left). The initial day of storage (0 tp) was used as the control group, wherein all conditions were clustered together with good fitness (0.971) and predictability (0.735). The targeted constituents were discriminated in the positive quadrant of the principle component t[1], which was initially dominant for all conditions.

However, on the 14th day (1 tp) of storage, some groups were distinguishable from the others. Principle component t[1] expressed 89% of total variation showing discrimination of the 1-time point of D on the positive and A on the negative quadrant with carvacrol and thymoquinone concentrations being relatively high in D. Although the composition of D decreased after the 1st day, the constituents were seen to be better preserved until the 28th day compared to the other conditions. The space in the vial flushed with the nitrogen gas might have assisted in the constituents' preservation. Meanwhile, at this time point, C could be distinguished from the others in the negative quadrant of the principle component t[2] with high content of 2-thujene,* p*-cymene, limonene, and longifolene. The model was expressed with good fitness (*R*^2^*X*) and high predictability (*Q*^2^) of 0.953 and 0.81, respectively.

On the 28th day of storage, groups A to D were distinguishable by t[1], explaining 89% of the total spectral variation with good fitness and predictability values of 0.995 and 0.956. The positive quadrant contained the observations of A and B, whereas C and D were in the opposite quadrant. The loading of biplots showed many variables present in the positive quadrant indicating that the samples stored in conditions C and D contained higher volatiles than A and B. Based on this observation, it is suggested that conditions C and D, in which the whole seeds were stored in a clear transparent vial and flushed with nitrogen gas, respectively, were better in preserving the volatile constituents, with D showing the higher stability since it has less intravariation among the replicates compared to C.

#### 3.3.2. Discrimination between Conditions E, F, and G (Ground Seeds)

The PCA biplots in Figure 5 (right) display discrimination among the ground seed samples E, F, and G. No clear distinction could be made on the 1st day of storage as all of the groups clustered together suggesting their sharing of similar constituent compositions. The score plot showed that these groups were concentrated at the center with principle component t[1] demonstrating 86% of total variation. The model was fit with *R*^2^*X* and *Q*^2^ values of 0.949 and 0.796, respectively. However, the intravariation among the F 0 tp replicates was higher due to the inconsistent exposure of the volatile constituents upon being crushed.

After 14 days of storage, there was clear separation between of E and F on the positive and G on the negative quadrant of t[1] with good fitness and predictability model (*R*^2^*X* cum = 0.919, *Q*^2^ cum = 0.722). The loading biplot showed all constituents were discriminated in the positive quadrant of t[1] wherein carvone, thymoquinone, carvacrol, and longifolene were dominant in F; meanwhile, 2-thujene and* p*-cymene were higher in E. The constituents were relatively low in G, which might be due to the flushed out volatiles with the nitrogen gas.

On the 28th day, F and G were on the left quadrant of t[1] with 90% of total variation, while E was on the right quadrant but showed high intravariation among its replicates. The loading biplot exhibited the fact that the content of the constituents was higher in E compared to the other two groups. This model was evaluated with a good fitness of 0.97 and a high predictability of 0.827. Among the three storage conditions for the ground seeds, E gave high concentrations of the metabolites, even after storage for 28 days.

#### 3.3.3. Suggested Pathways of the Volatile Constituent Changes

The major compounds of* N. sativa* essential oil and most other aromatic plants are of the terpenoid family [28]. Terpenes form a structurally and functionally diverse family of natural product constituents which are derived from head-to-tail combination of several 5-carbon-base (C_5_) units called isoprene. The common terpenes starting from the simplest one is hemiterpene (C_5_), followed by monoterpene (C_10_), sesquiterpene (C_15_), diterpene (C_20_), sesterterpene (C_25_), triterpene (C_30_), and tetraterpene (C_40_) [29, 30].

Botnick et al. (2012) studied the composition of the monoterpenes during the maturation of* N. sativa* seed [13] and proposed the biosynthetic pathway of the main volatile constituents (Supplementary Material 4) based on other previous studies of the Lamiaceae [31, 32]. It was suggested that the precursor geranyl diphosphate (GDP) was able to form *γ*-terpinene by cyclization before forming* p*-cymene by aromatization, which then produced carvacrol or thymol through hydroxylation. The formation of thymohydroquinone took place by another hydroxylation before turning into thymoquinone by oxidation. It was also suggested that thymoquinone could form dithymoquinone and higher oligocondesation products while under storage. Another monoterpene pathway concerns the formation of *α*-thujene and limonene directly from the precursor GPP. The limonene formed was then a precursor of carvone through further hydroxylation [33].

In the current study, the main monoterpene concentrations were observed to vary consistently during all of storage conditions which might be due to the constant changes of GDP, the basic precursor. The content of carvone and thymoquinone which are the monoterpene ketones decreased in storage condition E with the increment of carvacrol and longifolene. Percentage decrement of carvone was fast when the seeds were crushed. This is probably due to carvone's high volatility rate compared to the other constituents [17]. The decrement of thymoquinone could be explained by the formation of its derivative dithymoquinone and higher oligocondensation products [10]. In addition, the accumulation of carvacrol and* p*-cymene were also increased. As for the formation of the sesquiterpene longifolene, it depended on the cyclization of its starter precursor farnesyl pyrophosphate (FPP) [29].

## 4. Conclusions

The initial GC-MS analysis showed 25 identified significant volatiles in the ground seeds expressing higher relative amounts than the whole seeds. The compound stability under storage of the whole seeds was better compared to the ground seeds, since the volatiles were more exposed upon grinding (E, F, and G). Overall, all of the storage conditions did not give constant concentrations of the six major constituents, namely, 2-thujene,* p*-cymene, thymoquinone, carvone, carvacrol, and longifolene. All of the conditions experienced fluctuations of the constituent concentrations due to the four ecofactors of air, heat, and light. The seeds from either whole or ground material also played important roles in the stability of the* N. sativa* seeds. The findings of the present study exhibited that the conditions which showed minimal changes towards the environmental effects are when the whole seeds are stored in a transparent vial with nitrogen gas flush before storage. Among the whole seed samples of one-month storage, the fully filled clear vial sample (C) and the amber vial filled one-third flushed with nitrogen gas (D) with both kept in the dark were better conditions to retain the concentrations of the volatiles.

## Supplementary Material

The supplementary materials include the structures of Nigella sativa main constituents, the storage conditions which the seeds were grouped and stored, the peak areas obtained from GCMS to show the clear changes of those six selected main constituents, a proposed biosynthetic pathway which involves the biosynthesis of the main constituents, the GCMS of three incubation temperatures done before only one temperature was selected for further analysis, and GCMS for close comparison of phenol-4-methoxy-2,3,6-trimethyl and thymohydroquinone.

## Figures and Tables

**Table 1:** Volatile compounds detected in *N. sativa* whole and ground seeds by HS-GC/MS analysis.

Peak number	Compound	LRI^a^	LRI^b^	RT (min)	Peak area whole seed		Peak area ground seed	
					[×10^7^]	[%]	[×10^7^]	[%]
(1)	2-Thujene	927	930	6.31	81.95	7.85	2624.14	21.21
(2)	*α-*Pinene, (D)-	934	939	6.49	18.18	1.74	864.63	6.99
(3)	Sabinene	974	975	7.46	22.83	2.19	958.07	7.74
(4)	*β*-Pinene-(1S)-(−)	979	979	7.57	2.90	0.28	39.89	0.32
(5)	*p*-Cymene	1026	1025	8.81	259.63	24.86	3995.28	32.29
(6)	D-Limonene	1030	1029	8.92	33.37	3.20	428.32	3.46
(7)	*γ*-Terpinene	1059	1060	9.76	4.02	0.38	529.99	4.28
(8)	Terpinolene	1089	1089	10.57	4.19	0.40	29.67	0.24
(9)	*cis*-4-methoxy thujane^*∗*^	1098	—	10.79	8.17	0.78	200.61	1.62
(10)	*trans*-4-methoxy thujane^*∗*^	1121	—	11.43	34.48	3.30	777.74	6.29
(11)	1,3,8-*p*-Menthatriene	1139	1110	11.97	2.10	0.20	22.59	0.18
(12)	Pyranone^*∗*^	1151	—	12.18	2.56	0.25	5.90	0.05
(13)	Ethanone, 1-(1,4-dimethyl-3-cyclohexen-1-yl)-^*∗*^	1168	—	10.44	4.26	0.43	52.42	0.43
(14)	Terpinen-4-ol	1183	1177	13.07	6.91	0.66	69.29	0.56
(15)	*p*-Cymen-8-ol	1191	1183	13.60	3.23	0.31	17.00	0.14
(16)	*β*-Cyclocitral^*∗*^	1207	—	13.80	7.735	0.74	121.53	0.98
(17)	2,6-Octadiene,1-(1-ethoxyethoxy)-3,7-dimethyl-^*∗*^	1222	—	14.22	1.47	0.14	10.45	0.08
(18)	D-Carvone	1249	1243	14.92	171.45	16.42	37.03	0.30
(19)	Thymoquinone	1255	1252	15.22	180.19	17.25	1013.95	8.19
(20)	Bornyl acetate	1288	1289	13.60	8.94	0.86	19.70	0.16
(21)	Carvacrol	1311	1299	16.43	64.36	6.16	151.11	1.22
(22)	*α*-Longipinene	1357	1353	17.84	10.56	1.01	77.14	0.62
(23)	*α*-Copaene	1382	1377	18.47	8.36	0.80	12.16	0.10
(24)	Longifolene	1415	1408	19.31	42.86	4.10	239.10	1.93
(25)	Phenol, 4-methoxy-2,3,6-trimethyl-	1573	—	22.77	59.68	5.71	75.21	0.61

LRI^a^: acquired linear retention indices; RT: retention time.

^b^ [11]; ^*∗*^putative compounds.
