# Supplementary material for: Stability Study of Algerian Nigella sativa Seeds Stored under Different Conditions
Source: J Anal Methods Chem. 2017 Feb 1;2017:7891434. doi: 10.1155/2017/7891434 (PMC5309406; doi:10.1155/2017/7891434)
Supplement: Supplementary file 1 — The supplementary materials include the structures of Nigella sativa main constituents, the storage conditions which the seeds were grouped and stored, the peak areas obtained from GCMS to show the clear changes of those six selected main constituents, a proposed biosynthetic pathway which involves the biosynthesis of the main constituents, the GCMS of three incubation temperatures done before only one temperature was selected for further analysis, and GCMS for close comparison of phenol-4-methoxy-2,3,6-trimethyl and thymohydroquinone. [file 7891434.f1.pdf]

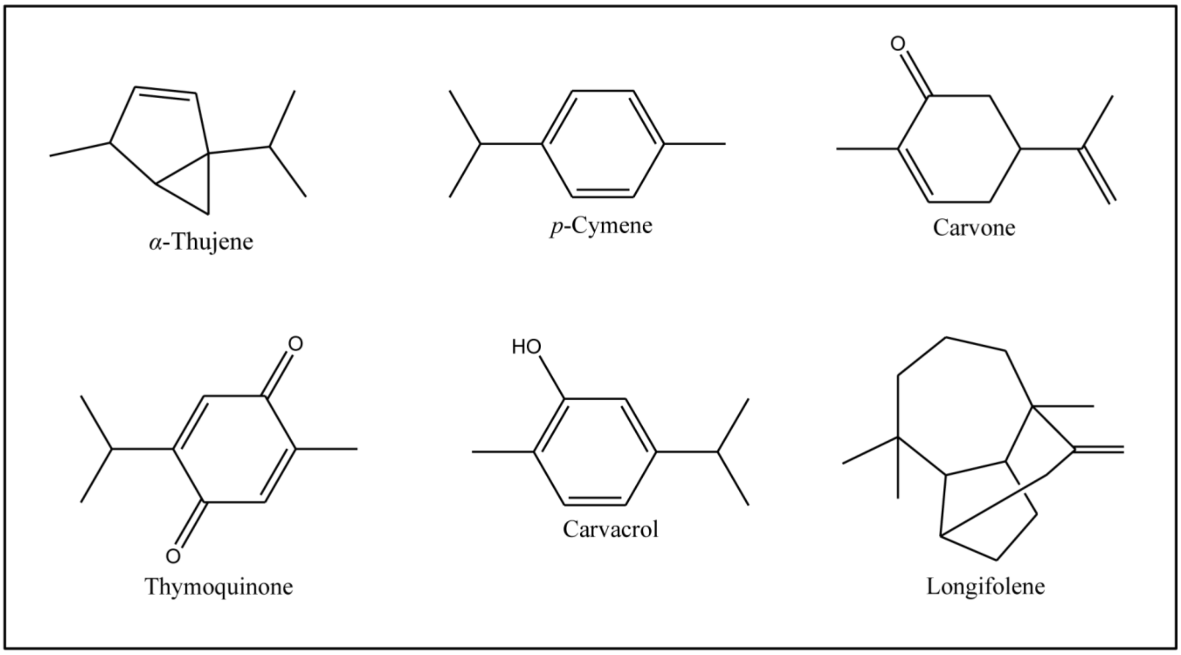

Supplementary Material 1 **Chemical structures of *N. sativa* seeds main constituents (source: Liu *et al*., 2011)**

***NIGELLA SATIVA* SEEDS**

- Whole seeds
- Ground seeds

CONDITION OF STORAGES

Condition	Whole seeds	Ground Seeds	Vial	Nitrogen	Light
A	1/3	-	Amber	-	-
B	Full	-	Amber	-	-
C	Full	-	Transparent	-	-
D	1/3	-	Amber	Flush	-
E	-	1/3	Amber	-	-
F	-	Full	Amber	-	-
G	-	1/3	Amber	Flush	-
H	1/3	-	Amber	-	Expose

STORAGE TIME POINTS

- 1 day
- 14 days
- 28 days

GC/MS ANALYSIS (HEAD SPACE)

MULTIVARIATE DATA ANALYSIS (MVDA)

Supplementary Material 2 Flowchart of eight storage conditions (A to H)

Supplementary Material 3 Peak area (10x^{7}) of selected constituents

Storage time	Peak area of constituents (10x^{7})					
	α- Thujene	p-Cymene	Carvone	Thymoquinone	Carvacrol	Longifolene
**Condition A**						
A0 (1st day)	6.617±1.074^{a}	26.239±3.638^{a}	8.041±1.363^{a}	10.535±2.100^{a}	6.292±0.980^{a}	2.967±0.441^{a}
A1 (14th day)	3.233±0.517^{b}	12.542±1.540^{b}	4.235±0.628^{b}	5.062±0.928^{b}	4.426±1.201^{b}	1.561±0.222^{b}
A2 (28th day)	8.782±0.122^{c}	2.682±0.244^{c}	0.820±0.094^{c}	0.981±0.139^{c}	8.880±0.078^{c}	0.446±0.094^{c}
**Condition B**						
B0 (1st day)	6.348±0.820^{a}	26.297±4.633^{a}	11.216±4.811^{a}	12.130±4.198^{a}	11.828±2.453^{a}	3.963±1.678^{a}
B1 (14th day)	4.821±1.982^{a}	20.999±8.988^{a}	6.544±2.751^{a}	6.934±2.738^{b}	7.851±2.958^{b}	2.660±1.151^{a}
B2 (28th day)	0.809±0.240^{b}	2.541±0.703^{b}	0.766±0.279^{b}	0.764±0.209^{c}	0.644±0.125^{c}	0.401±0.099^{b}
**Condition C**						
C0 (1st day)	5.969±2.128^{a}	23.006±4.940^{a}	7.486±1.333^{a}	9.140±1.407^{a}	6.015±1.738^{a}	2.642±0.471^{ab}
C1 (14th day)	6.752±2.560^{a}	26.297±13.758^{a}	9.697±8.936^{a}	7.380±5.127^{a}	7.257±5.236^{a}	3.812±2.864^{b}
C2 (28th day)	11.826±7.063^{b}	52.429±13.802^{b}	27.798±15.660^{b}	22.436±14.947^{a}	19.279±9.459^{b}	7.394±2.735^{bc}
**Condition D**						
D0 (1st day)	6.797±0.899^{a}	25.625±2.935^{a}	8.906±1.104^{a}	7.053±1.289^{a}	14.659±1.237^{a}	3.501±0.399^{a}
D1 (14th day)	7.885±0.828^{a}	29.919±4.001^{a}	10.787±3.753^{a}	12.958±4.182^{a}	13.291±3.866^{a}	4.285±1.464^{a}

D2 (28th day)	13.018±1.179^{b}	49.105±4.136^{b}	16.123±1.216^{b}	32.128±11.737^{b}	16.248±1.387^{a}	6.180±0.423^{b}
**Condition E**						
E0 (1st day)	938.035±1241.148^{a}	1831.768±1922.327^{a}	4.249±4.046^{a}	42.947±63.688^{a}	10.323±8.767^{a}	24.653±24.701^{a}
E1 (14th day)	1235.740±201.471^{a}	3922.320±605.098^{a}	2.084±0.555^{a}	24.652±2.479^{a}	8.656±2.758^{a}	18.800±7.039^{a}
E2 (28th day)	1363.585±843.986^{a}	4304.681±2294.927^{a}	2.975±1.739^{a}	37.720±22.497^{a}	15.199±8.119^{a}	30.582±16.626^{a}
**Condition F**						
F0 (1st day)	702.425±943.191^{ab}	2408.180±2427.564^{ab}	10.214±4.864^{ab}	84.896±94.554^{a}	22.714±18.898^{ab}	51.315±44.861^{ab}
F1 (14th day)	1359.189±820.535^{b}	4074.124±1111.216^{b}	4.764±1.238^{b}	42.108±16.200^{a}	19.330±5.179^{b}	42.954±12.033^{b}
F2 (28th day)	77.092±158.586^{ac}	480.307±891.473^{ac}	1.659±2.481^{bc}	8.220±14.150^{a}	1.923±1.668^{bc}	3.632±3.482^{bc}
**Condition G**						
G0 (1st day)	22.786±26.056^{a}	1141.381±850.809^{a}	3.055±0.512^{a}	70.464±43.856^{a}	18.257±4.669^{a}	20.742±4.972^{a}
G1 (14th day)	2.915±4.695^{a}	110.938±167.432^{b}	0.590±0.168^{b}	23.399±5.982^{b}	2.783±1.662^{b}	4.286±2.267^{b}
G2 (28th day)	0.561±1.203^{a}	10.916±19.501^{b}	0.015±0.044^{c}	0.428±0.204^{b}	0.038±0.017^{b}	0.159±0.066^{b}
**Condition H**						
H0 (1st day)	2.885±0.097^{a}	8.209±0.212^{a}	3.393±1.697^{a}	5.561±2.774^{a}	5.421±1.123^{a}	2.170±0.261^{a}
H1 (14th day)	0.020±0.005^{b}	0.051±0.008^{b}	0.082±0.009^{b}	0.096±0.010^{b}	0.035±0.004^{b}	0.037±0.003^{b}
H2 (28th day)	1.828±0.430^{c}	0.247±0.080^{b}	0.391±0.137^{b}	0.460±0.139^{b}	0.294±0.108^{b}	0.274±0.110^{b}

* Mean values with similar letters within columns for each condition of storages are not significantly different among the storage time points (Turkey comparison test, $P < 0.05$)

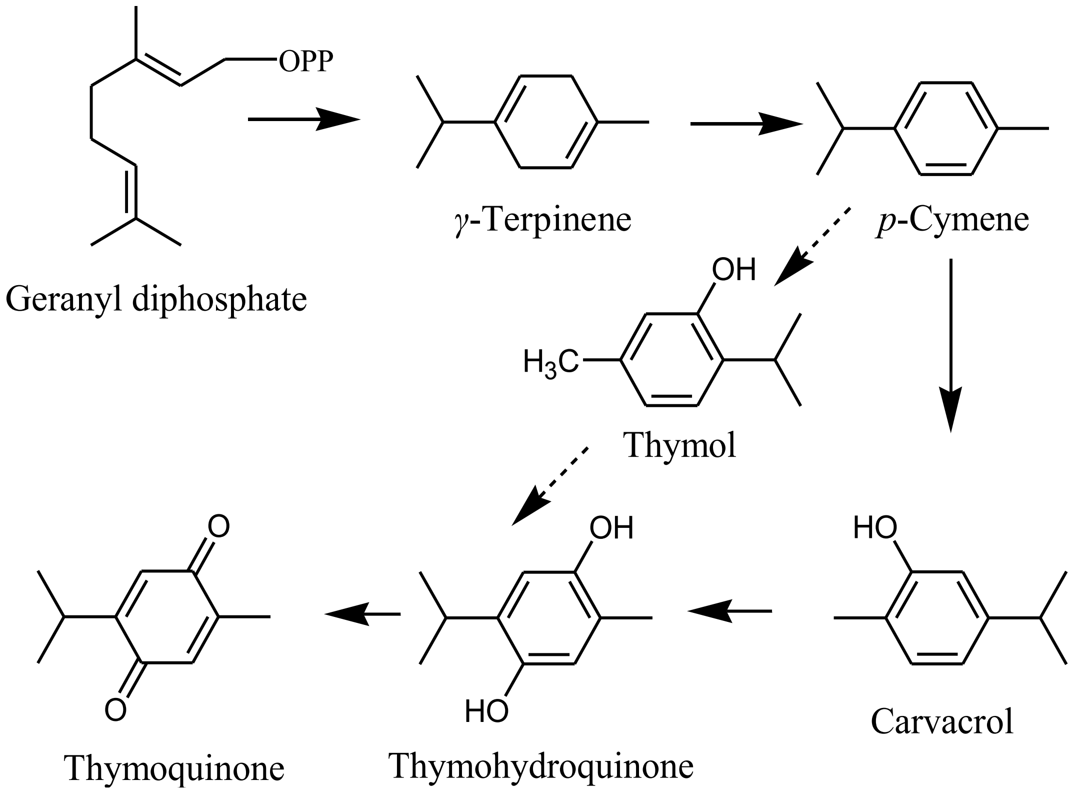

Supplementary Material 4 Biosynthetic pathway of main of main constituents in *N. sativa* (source : Botnick et al., 2012)

Incubation temperature 80 degree

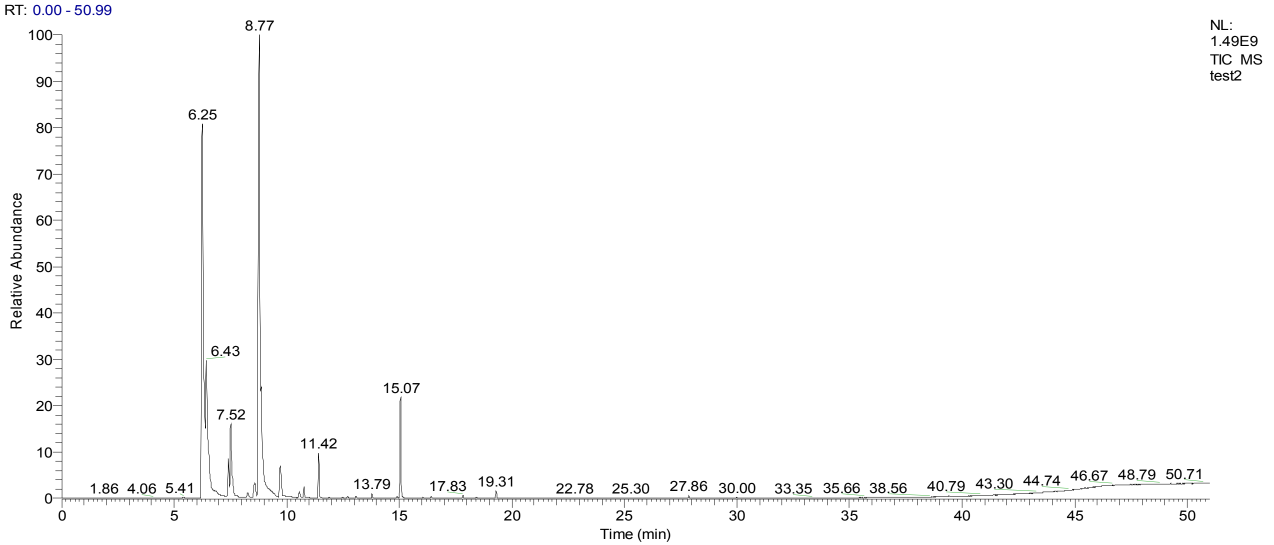

Incubation temperature 100 degree

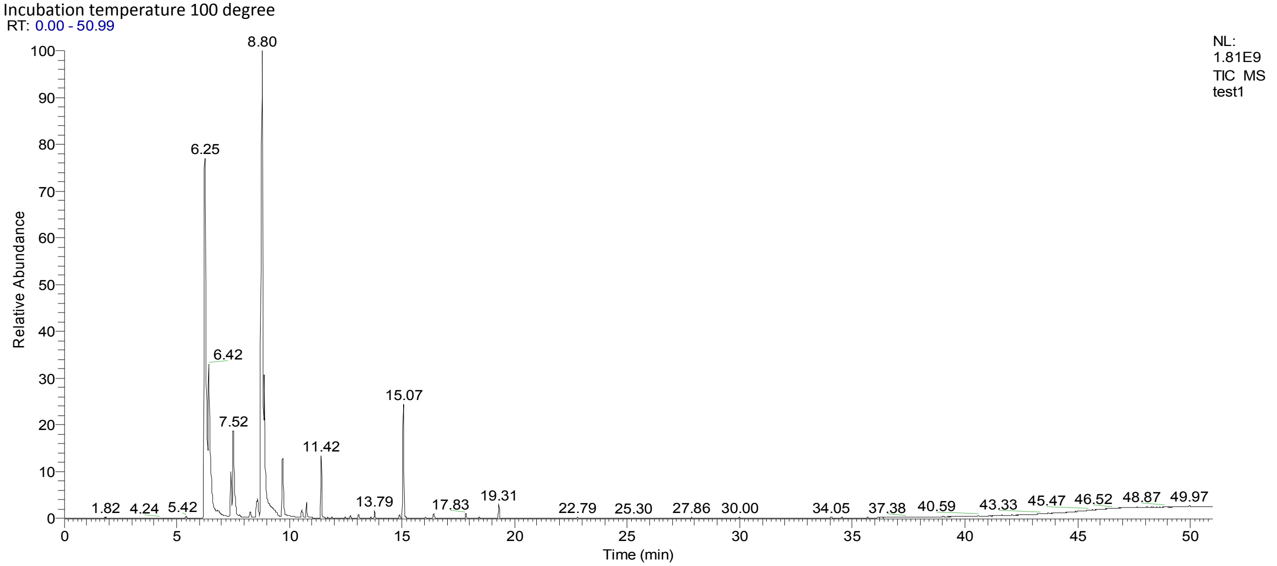

Incubation temperature 160 degree

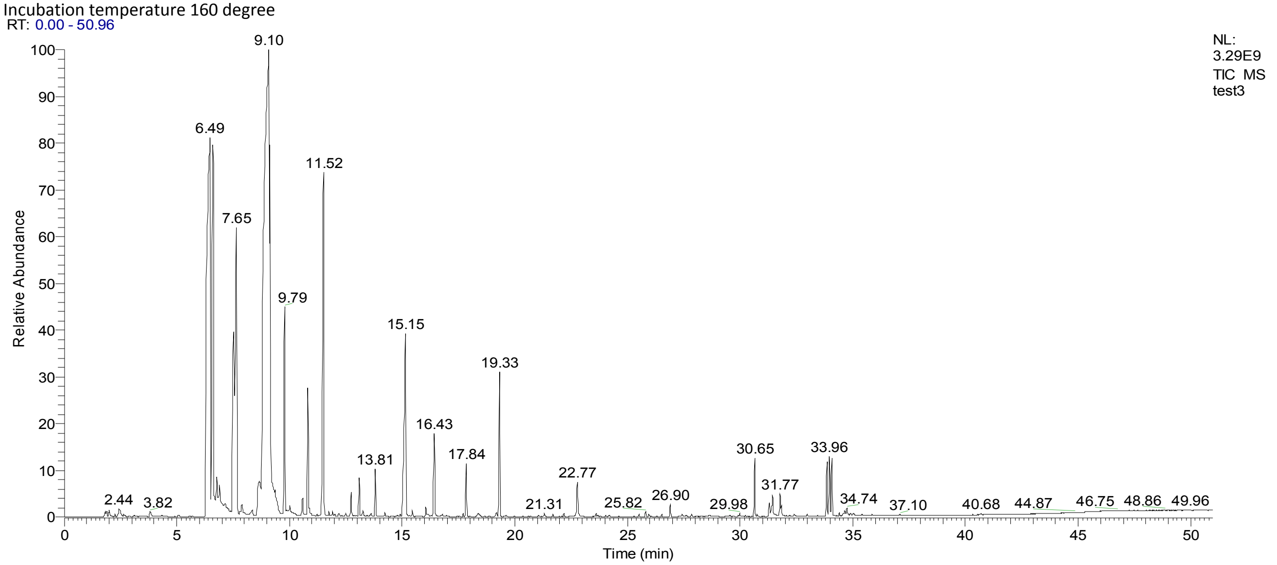

Supplementary Material 5 Result of peak area from three different incubation temperature

Mass spectrum of thymohydroquinone detected in Flavour and Fragrance Natural and Synthetic Compounds (FFNSC) version 1.3 library

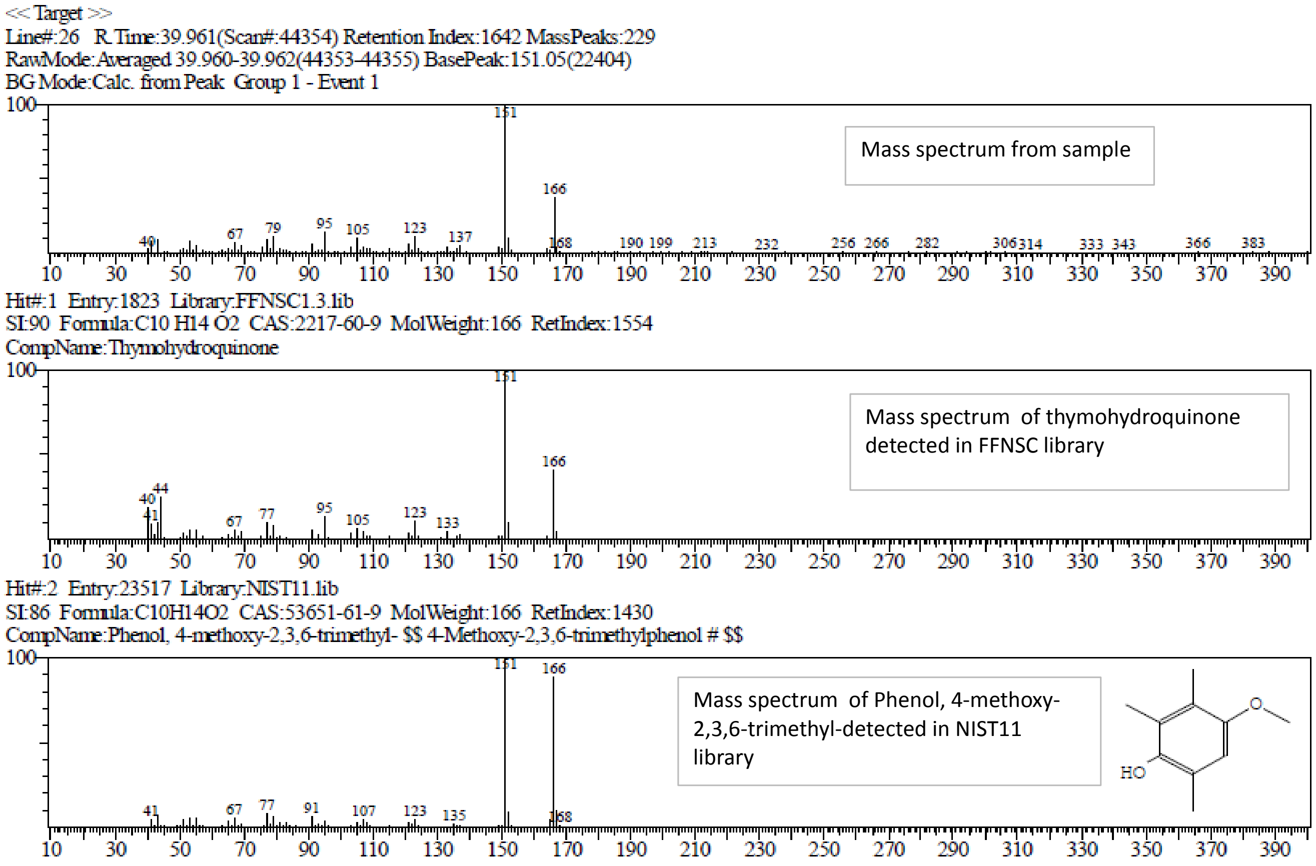

Supplementary Material 6 The molecular weights and mass spectra of phenol,4-methoxy-2,3,6-trimethyl and thymohydroquinone
